# Annotations

**Published:** 1900-05-19

**Authors:** 


### Annotations.

### The "Boycott" of a Congress.

The past and present teachers of gynæcology and obstetrics in the metropolitan medical schools now practising in London have decided with quite an interesting unanimity to boycott the "fourth International Congress of Gynæcology and Obstetrics" which, as is announced, it has been decided to hold in London in 1902, under the presidency of Professor Simpson, of Edinburgh. This they do in sorrow, as a protest, not against the distinguished professor, but against the manner and source of the invitation, and in view of the present disjointed condition of the medical profession it is somewhat instructive to note the sort of thing which is capable of producing an absolutely unanimous action in any group among its members, an action so unanimous in this case that the letter in which the boycott was announced was, we believe, signed by every member of the set. Of course, we quite understand their feeling that the source the invitation came from is hardly a fount of knowledge to which gynæcologists from all parts of the world can be expected to rush in great numbers, and that for a group of specialists outside the sacrosanct circle of the metropolitan teachers to lay so big an egg as the "fourth International Congress of Gynæcology and Obstetrics" is an unheard-of piece of impertinence. Indeed, we quite agree that, in the words of the boycott, those who issued the famous invitation "have assumed a representative position to which they are . . . not entitled." On that side of the question we make no comment. All we do is to point to the interesting psychological fact that, difficult as it is to obtain anything like united action among medical men for any great object, there are ways of stimulating certain groups of them to absolute unanimity. That an international congress should be held in London and should be "bossed" by the non-elect was too much to be borne.

### Legislation by Definition.

In the framing of an Act of Parliament which is held to be of vital importance to the lives and fortunes of a large number of our fellow-subjects, it would seem not unreasonable to expect that its provisions should be expressed clearly, clause by clause, in the body of the Act, and in words which could not be disputed. Some people, however, do not appear to see the matter in this light, and prefer to put deep and hidden meanings into definitions, and leave the lawyers afterwards to fight the matter out. As if the difficulties in the way of legislating for the midwives were not already great enough, there is a party—strongly represented in the British Medical Association—which persists in complicating the whole affair by insisting on what we cannot but regard as a fancy definition of the midwife. What is a midwife? What is the person for the regulation of whose conduct legislation is proposed? According to the Bill now before Parliament she is "a woman who undertakes for reward to attend women in childbirth where no medical practitioner is engaged or employed." This exactly describes the person aimed at, and that surely ought to be enough. But the deputation from the Council of the British Medical Association which waited upon the Solicitor-General last Friday is not content with a descriptive definition, however accurate. What they want to do is to legislate for the midwife's conduct by the definition they give of her trade, and they ask that the term midwife shall be defined as meaning "a woman who undertakes for gain to attend women during natural labour and the lying-in period," the object of this very disputatious definition being to prevent the midwife regarding herself as an independent practitioner, and to prevent her from retiring from the case after the birth of the child, and leaving the nursing in the hands of someone else. What is plain, however, is that in grasping at the shadow they lose the substance, and that in endeavouring to pass a restrictive measure by means of a few words of definition, instead of by a separate clause in the body of the Bill, they spoil the whole measure, leaving outside of and beyond its scope the very people whom it is most important to bring most strictly under its regulations. In the face of all the anxieties involved in the present position of affairs, it is distressing to find that when at last the British Medical Association has obtained a hearing, this is the sort of drivel with which its deputation entertains the Solicitor-General.

### The Failure of the Filters.

The terrible list of deaths from enteric fever issued by the War Office last Monday shows that, notwithstanding the accuracy of our knowledge of the pathology of this disease and the complete grip which we have of the whole question so far as theory is concerned, our measures of defence against this scourge of armies are as yet practically in their infancy. During the last two or three years there is no doubt that our eyes have been considerably opened in regard to the various modes in which this disease may be and often is distributed, and when all this newer knowledge gets thoroughly assimilated perhaps some fresh light may break in upon the subject. So far, however, as one may venture to speculate at a distance, and to speak on general principles, it seems extremely probable that so great an outburst as evidently is raging at the present time at Bloemfontein has originated in the well-recognised way as the consequence of pollution of the drinking water. Polluted water will probably always remain the principal source of infection where typhoid occurs in great epidemics; and, unfortunately, much of the knowledge which has been gained in recent years about filtration has tended to throw doubt upon the power of ordinary filters to produce a safe and drinkable water. Indeed, a sort of despair has seized upon scientific men upon this subject. We are, however, by no means sure that the last word has been said upon the purification of water by mechanical and, perhaps, by chemical means. Bacteriologists have set up so high a standard as to have condemned off-hand all the old-fashioned filters, and have given us instead some very beautiful and perfect arrangements, which, however, clog up directly with South African mud. But we are beginning to appreciate the biology of the subject, and to understand what a very tender organism the typhoid bacillus really is; and it is on the cards that some better way of getting rid of these microbes may be devised than the apparently simple but really very difficult method of straining them out.

